# The effect of synaptic plasticity on orientation selectivity in a balanced model of primary visual cortex

**DOI:** 10.3389/fncir.2015.00042

**Published:** 2015-08-20

**Authors:** Soledad Gonzalo Cogno, Germán Mato

**Affiliations:** Comisión Nacional de Energía Atómica and Consejo Nacional de Investigaciones Científicas y Técnicas, Centro Atómico Bariloche and Instituto BalseiroBariloche, Argentina

**Keywords:** plasticity, orientation selectivity, visual cortex, orientation map, synaptic reconnection

## Abstract

Orientation selectivity is ubiquitous in the primary visual cortex (V1) of mammals. In cats and monkeys, V1 displays spatially ordered maps of orientation preference. Instead, in mice, squirrels, and rats, orientation selective neurons in V1 are not spatially organized, giving rise to a seemingly random pattern usually referred to as a salt-and-pepper layout. The fact that such different organizations can sharpen orientation tuning leads to question the structural role of the intracortical connections; specifically the influence of plasticity and the generation of functional connectivity. In this work, we analyze the effect of plasticity processes on orientation selectivity for both scenarios. We study a computational model of layer 2/3 and a reduced one-dimensional model of orientation selective neurons, both in the balanced state. We analyze two plasticity mechanisms. The first one involves spike-timing dependent plasticity (STDP), while the second one considers the reconnection of the interactions according to the preferred orientations of the neurons. We find that under certain conditions STDP can indeed improve selectivity but it works in a somehow unexpected way, that is, effectively decreasing the modulated part of the intracortical connectivity as compared to the non-modulated part of it. For the reconnection mechanism we find that increasing functional connectivity leads, in fact, to a decrease in orientation selectivity if the network is in a stable balanced state. Both counterintuitive results are a consequence of the dynamics of the balanced state. We also find that selectivity can increase due to a reconnection process if the resulting connections give rise to an unstable balanced state. We compare these findings with recent experimental results.

## Introduction

Neurons in primary visual cortex are characterized by being selective to several stimulus features, such as orientation, ocular dominance or retinotopy. One of the most interesting aspects of the primary visual cortex is that these cortical features can be spatially organized, i.e., nearby neurons tend to have similar optimal stimuli. One important receptive field property, such as orientation preference, can be mapped rather smoothly across the cortical surface. This was found in cats in Hubel and Wiesel (1961, 1962), Bonhoeffer and Grinvald (1991) and also confirmed in primates (Wiesel and Hubel, 1974). For these systems we say that an orientation map is present.

More recently it was found that in rodents the visual cortex behaves in a very different way. Cells in the primary visual cortex are selective to orientation (Dräger, 1975; Scholl et al., 2013) but there are no orientation maps (Ohki et al., 2005; van Hooser et al., 2005). In fact, neighboring cells display completely different preferred orientations, giving rise to the salt-and-pepper organization of orientation selectivity (Ohki et al., 2007). This structure can appear even for highly visual animals (van Hooser et al., 2005).

Having high degree of orientation selectivity for such different structures leads to question the structural organization of the intracortical connections. It is known that neurons tend to be connected with other neurons in their neighborhood (Holmgren et al., 2003; Stepanyants et al., 2008); for systems with columnar organization this means neurons with similar selectivity properties. However, for the salt-and-pepper structure the situation is not so clear. Neurons with similar response properties could be more strongly connected independently of the physical distance (Ko et al., 2011; Cossell et al., 2015). Moreover, this functional connectivity could be the result of plasticity processes. For instance, in Ko et al. (2013) it was found that functional microcircuits are generated during development, but their presence leads only to a small increment of selectivity.

From a theoretical point of view, it was recently found that even if the connectivity patterns are totally random, selectivity can be maintained from layer 4 to layer 2/3 (Hansel and van Vreeswijk, 2012). This happens because as the network is populated by both excitatory and inhibitory neurons operating in a balanced activity regime, untuned excitatory and inhibitory inputs roughly cancel each other giving rise to a sizable net tuned input. The theory of balanced state has been proposed to explain the temporal variability of the response in systems that are highly connected (Softky and Koch, 1993). This theory explains the variability without doing fine tuning of parameters (van Vreeswijk and Sompolinsky, 1998) and was later supported by experimental works (Destexhe et al., 2003; Shu et al., 2003; Haider et al., 2006).

Even if balanced networks with totally random connections can display a substantial degree of selectivity, it has been suggested (Corey and Scholl, 2012) that increasing the connection probability of nearby neurons with similar orientation preference might further enhance their selectivity. This increment could be generated by plasticity processes at the synaptic level.

Here we intend to clarify the problem of the effect of plasticity on orientation selectivity. The questions we address are: under which conditions can plasticity improve selectivity? Is the effect of plasticity different for the salt-and-pepper organization and for systems with orientation maps? If plasticity does improve selectivity, what is the main mechanism responsible for that change?

We study these questions by analyzing a computational model of layer 2/3 and a reduced one-dimensional model of orientation selective neurons. Since we are modeling cortical activity (characterized by both highly irregular response and large connectivity), our computational and reduced models are in the balanced state. We analyze two plasticity mechanisms, one of them involves spike-timing dependent plasticity (STDP) and the other considers the reconnection of neuronal interactions according to the preferred orientations of the pre- and post-synaptic neurons.

We find that spike-timing dependent plasticity improves selectivity but it works in a somehow unexpected way, that is effectively decreasing the modulated part of the intracortical connectivity as compared to its non-modulated part. We find this conclusion to be valid both for systems with salt-and-pepper organization and with orientation maps. The effect of reconnection of interactions is also non-intuitive: As predicted by the one dimensional model, if the network dynamics is in a stable balanced state, functional connectivity leads to a decrease in selectivity. However, if the balanced state is unstable, functional connectivity can indeed improve selectivity.

## Materials and methods

### The model network

**Figure 1:**
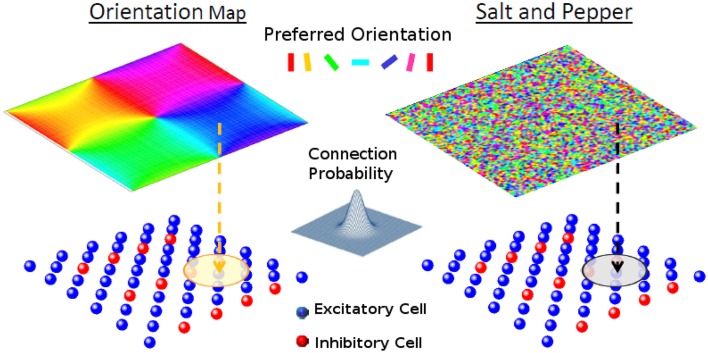
**The network model**. Each one of the cells of layer 2/3 (lower panels) receives a feed-forward input whose preferred orientation is shown in the corresponding position of the upper panel. The circle in layer 2/3 represents the width of the recurrent connectivity matrices. **Left:** orientation map; **Right:** salt-and-pepper layout.

The model is composed of one layer representing a square patch of layer 2/3 of size *M* × *M*, where we assume *M* to be approximately 1 mm. It has *N*_*E*_ excitatory and *N*_*I*_ inhibitory neurons (see Figure 1). We denote neuron *i* = 1, …, *N*_*A*_ of population *A* = *E, I*, with neuron (*i, A*). The neurons are arranged on a square grid and the position (*x*_*iA*_, *y*_*iA*_) of neuron (*i, A*) in its layer is given by $x_{iA}=i_{x}M∕\sqrt{N_{A}},y_{iA}=i_{y}M∕\sqrt{N_{A}}$, where $i_{x}=\left(i-1\right)\;\mathrm{mod}\;\sqrt{N_{A}}$ and $i_{y}=\left\lfloor \left(i-1\right)∕\sqrt{N_{A}}\right\rfloor$. Here, ⌊*x*⌋ is the largest integer equal or smaller than *x*. We choose *N*_*A*_ in such a way that $\sqrt{N_{A}}$ is an integer number. Thus, indices *i*_*x*_ and *i*_*y*_ go from 0 to $\sqrt{N_{A}}-1$. Unless otherwise specified in the simulations presented here, we take *N*_*E*_ = 8100 and *N*_*I*_ = 2025.

### Single neuron dynamics of layer 2/3 cells

Neurons in layer 2/3 are described by a leaky integrate-and-fire model. The membrane potential *V*_*iA*_ of neuron (*i, A*), *A* = *E, I*, evolves in time according to:

(1) $$\begin{array}{l} \tau \frac{dV_{iA}}{dt}=-V_{iA}+R_{m}\left(I_{L4,iA}+I_{rec,iA}+I_{back,iA}\right), \end{array}$$

where τ is the membrane time constant, *R*_*m*_ the membrane resistance (see Section Default Parameters), *I*_*L*4, *iA*_ is the input current from layer 4, *I*_*rec, iA*_ is the recurrent input from all the other neurons in layer 2/3 and *I*_*back, iA*_ represents a background input from other cortical regions. When the membrane potential reached the threshold value *V*_*T*_ = 30 mV, it was immediately reset to *V*_*rest*_ = 0 mV.

### The model of the feed-forward input

The input is considered to have a firing rate that depends on the visual stimulus, which is assumed to be a sinusoidal grating with a fixed wavelength. For a grating with orientation θ, the input to neuron (*i, A*) is given by:

(2) $$I_{L4,iA}=g_{AL}K_{AL}\;fr_{L}(1+2\rho_{iA}\cos(2(\theta -\theta_{iA})),$$

where *g*_*AL*_ is the individual efficacy of the feed-forward synapse, *K*_*AL*_ is the number of feed-forward synapses, *fr*_*L*_ is the average firing rate of cells in layer 4, ρ_*iA*_ is the effective modulation of the feed-forward input with respect to the orientation of the stimulus and θ_*iA*_ is the stimulus orientation for which the feed-forward input to neuron (*i, A*) takes its maximal value. In the following we assume that all the neurons in each population receive inputs with the same modulation, i.e., ρ_*iA*_ = ρ_*A*_.

We analyze two different possibilities for the orientations θ_*iA*_:

1. *Random orientations.* The values of θ_*iA*_ are independent random variables with uniform distribution between 0 and π. This is known as the salt-and-pepper distribution.
2. *Continuous orientation map.* The values of the optimal feed-forward inputs are given by:

(3) $$\begin{array}{rcl} \theta_{iA} & = & \arctan\left(\sin\left(2\pi y_{iA}∕M\right)∕\sin\left(2\pi x_{iA}∕M\right)\right)∕2+\\ \; & \; & \pi ∕2+\pi \left(1+sign\left(x_{iA}∕M-0.5\right)\right)∕4. \end{array}$$

This condition guarantees a continuous distribution of the optimal feed-forward inputs (except at the pinwheels) while respecting periodic boundary conditions see Figure 1.

### Recurrent interactions

We assume that the recurrent interactions in layer 2/3 are random. The probability of connection between neuron (*j, B*) and (*i, A*) (*A, B* = *E, I*) is given by:

(4) $$\begin{array}{l} p_{iA,jB}=Z_{AB}G\left(x_{iA}-x_{jB},\sigma_{AB}\right)G\left(y_{iA}-y_{jB},\sigma_{AB}\right) \end{array}$$

where *G* is the *periodic* Gaussian with period *M*, $G\left(x,\sigma \right)=\overset{\infty }{\underset{k\;=\;-\infty }{\sum }}\exp\left(-{\left(x-Mk\right)}^{2}∕\left(2\sigma^{2}\right)\right)$. *Z*_*AB*_ insures that the average number of connections from population *B* to population *A* is given by *K*_*AB*_. In the following we take *K*_*AB*_ = *K*.

In principle, the probability of having a connection depends on the distance between the pre- and-postsynaptic neurons (Holmgren et al., 2003; Stepanyants et al., 2008). For the system with an orientation map, however, nearby neurons tend to have similar preferred inputs. This implies that we necessarily have functional connectivity, i.e., neurons with similar feed-forward inputs are more likely to be connected. On the other hand for the salt-and-pepper structure there is no correlation between the position and the preferred stimulus. Functional connectivity can be introduced by reconnecting neurons with similar preferred orientations (see Section Reconnection Probability).

### Synaptic currents

After the connection probabilities are evaluated the connectivity matrices *C*^*AB*^ (*A, B* = *E, I*) can be determined. Their elements, $C_{ij}^{AB}$, are 0 or 1 according to the probabilities of Equation (4).

The recurrent currents are given by:

(5) $$\begin{array}{l} I_{rec,iA}=\underset{B}{\sum }g_{AB}\overset{N_{B}}{\underset{j\;=\;1}{\sum }}w_{iA,jB}C_{ij}^{AB}\underset{k}{\sum }\frac{\exp\left(-\left(t-t_{k,j}\right)∕\tau_{syn,B}\right)}{\tau_{syn,B}}, \end{array}$$

where *t*_*k, j*_ is the time of the *k*^*th*^ spike of presynaptic neuron *j*. The time scale of the synaptic interactions is controlled by τ_*syn, A*_ and their strength is determined by the coupling parameters *g*_*AB*_ and by the normalized synaptic efficacies *w*_*iA, jB*_. These parameters are set to 1 at the beginning of the simulation and can be modified in the presence of synaptic plasticity (see Section Spike-timing Dependent Plasticity).

Let us note that, in order to have a well defined scaling in the limit of large connectivity, while keeping a highly variable temporal activity, the couplings have to be scaled with the inverse of the square root of the connectivity (van Vreeswijk and Sompolinsky, 1996, 1998). For that reason we define $g_{AB}=\frac{G_{AB}}{\sqrt{K_{AB}}}$ (*A, B* = *E, I*).

For the background current we use the expression:

(6) $$\begin{array}{l} I_{back,iA}=\sqrt{K}I_{back,A}. \end{array}$$

This represents the effect of *K* excitatory only inputs, each one with synaptic strength that scales as $1∕\sqrt{K}$.

### Numerical procedures and analysis of the results

The numerical simulations were performed using an Euler scheme to integrate the neuronal dynamics, the neuronal interactions and the synaptic plasticity (Press et al., 1992). The time step is δ*t* = 0.05 ms. The typical simulation run was 20 s long and the transient period was 0.1 s. The transient was chosen in order to let the firing rate stabilize after the beginning of the simulation.

For stimulus angle θ the firing rate of the neuron was estimated by averaging its spike response over the whole run (after the transient). We took 9 stimulus angles: θ = 0, 20, 40, 60, 80, 100, 120, 140, 160°. The selectivity was quantified using the Orientation Selectivity Index (OSI) of the activity:

(7) $$OSI_{iA}=\frac{\sqrt{{\left(\sum_{\theta }f_{iA}(\theta )\cos(2\theta )\right)}^{2}+{\left(\sum_{\theta }f_{iA}(\theta )\sin(2\theta )\right)}^{2}}}{\sum_{\theta }f_{iA}(\theta )},$$

where *f*_*iA*_(θ) is the firing rate of neuron (*i, A*) when the stimulus angle is θ. Notice that this definition of the orientation selectivity index corresponds to the ratio between the first and the zeroth Fourier components of the firing rate. This number goes between 0 and 1. It is 0 for flat tuning curves and becomes 1 when the tuning curve is a delta function.

The preferred orientation of neurons in layer 2/3 was estimated using the population vector. The angle of the population vector *PO*_*iA*_ of neuron (*i, A*) indicates its preferred orientation and is evaluated according to:

(8) $$\tan(2PO_{iA})=\frac{\sum_{\theta }f_{iA}(\theta )\sin(2\theta )}{\sum_{\theta }f_{iA}(\theta )\cos(2\theta )}.$$

To check that the time step in the numerical simulations was sufficiently small, we performed several simulations with a smaller time step, δ*t* = 0.025 ms, and verified that the results were not affected. We also performed some simulations on networks with approximately twice as many neurons (*N*_*E*_ = 16129, *N*_*I*_ = 4096), keeping the average number of connections into excitatory and inhibitory cells the same, to verify that our results were not an artifact of a small network size.

### Spike-timing dependent plasticity

**Figure 2:**
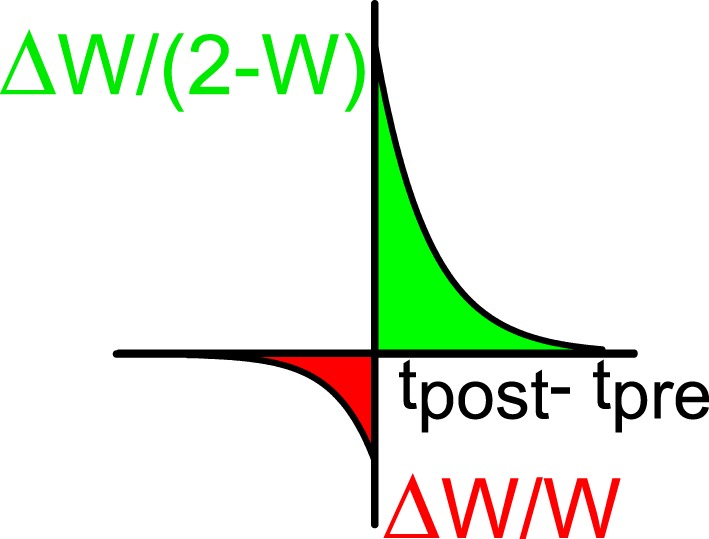
**STDP window: the relative changes of synaptic efficacy depend on the time difference between the post-synaptic (*t*_*post*_) and the pre-synaptic (*t*_*pre*_) spikes**. Green indicates facilitation and red depression.

The excitatory synapses undergo a process of plasticity according to the following rule (Bi and Poo, 1998; Abbott and Nelson, 2000). When neuron *j* in population *E* generates a spike at time *t*_*pre*_ and neuron *i* in population *A* (*A* = *E, I*) (which is connected post-synaptically to (*j, E*)) generates a spike at time *t*_*post*_, the normalized synaptic efficacy between the two neurons is modified according to:

(9) $$\begin{array}{l} w_{iA,jE}=w_{iA,jE}+\Delta w_{iA,jE}, \end{array}$$

where

(10) $$\begin{array}{l} \Delta w_{iA,jE}=a_{+}\exp\left(-\left(t_{post}-t_{pre}\right)∕\tau_{+}\right)\left(2-w_{iA,jE}\right) \end{array}$$

if *t*_*post*_ > *t*_*pre*_ or

(11) $$\begin{array}{l} \Delta w_{iA,jE}=a_{-}\exp\left(-\left(t_{pre}-t_{post}\right)∕\tau_{-}\right)w_{iA,jE} \end{array}$$

if *t*_*post*_ < *t*_*pre*_. We take *a*_+_ > 0, *a*_−_ < 0 (see Figure 2). This implies that if the postsynaptic spike comes after the presynaptic spike the connection becomes stronger and for the reverse order it becomes weaker. The multiplicative dependencies (Rubin et al., 2001) of the synaptic modification on the synaptic strengths (2 − *w*_*iA, jE*_ in Equation 10 and *w*_*iA, jE*_ in Equation 11) insure that the modifications become very weak as *w*_*ij*_ approach the bounds 0 or 2. Let us note that inhibitory synapses are not plastic, i.e., *w*_*iA, jI*_ is always equal to 1. The plasticity mechanism is applied during the first 2/3 of the simulation (13.33 s). During the last third of the simulation (6.66 s) the plasticity process is stopped and the activity is recorded in order to evaluate the selectivity properties and other parameters such as the values of the synaptic efficacies. During the whole simulation, only one stimulus orientation is applied.

### Reconnection probability

In the way it is described above, spike-timing dependent plasticity can give rise to the strengthening or weakening of the synaptic connections but not to a change in the connection probability (i.e., it does not create nor destroy connections). However, this change is observed in rodent visual cortex in Ko et al. (2013). Thus, in order to implement it, we first measure the preferred orientation of each neuron according to the population vector (see Equation 8). Then the probability of connection between neuron (*j, B*) and neuron (*i, A*) is given by:

(12) $${\hat{p}}_{iA,jB}=p_{iA,jB}(1+\epsilon_{cB}\cos(2(PO_{jB}-PO_{iA})),$$

where *p*_*iA, jB*_ is given by Equation (4). If parameter ϵ_*cB*_ > 0, neurons with similar preferred orientations are more likely to be connected than neurons with orthogonal preferred orientations. We assume that the reconnection parameter depends only on the presynaptic population. After the reconnection process is applied, the simulation continues for 6.66 s to measure the selectivity properties and the preferred orientations of the neurons. As before, only one stimulus angle is presented during each simulation.

### Default parameters

The membrane time constant is τ = 20 ms (Somers et al., 1995) and the membrane resistance is *R*_*m*_ = 38.3 MΩ (Koch, 1999). The parameters of the synapses are *G*_*EE*_ = 32, *G*_*EI*_ = −96, *G*_*IE*_ = 96, *G*_*II*_ = −128, *g*_*EL*_ = 1.65, *g*_*IL*_ = 1.65 nA ms, τ_*syn, E*_ = 25 ms, τ_*syn, I*_ = 4 ms (Hansel and van Vreeswijk, 2012). We choose a longer time constant for the excitation to represent a mixture of AMPA and NMDA synapses. Unless otherwise specified, the average recurrent connectivity is *K* = 500 and the feed-forward connectivity is *K*_*AL*_ = 250. The standard deviations of the recurrent connections are σ_*AB*_ = σ_*AL*_ = 0.2*M* (*A* = *E, I*). The firing rate of cells in layer 4 is *fr*_*L*_ = 15 Hz and the default values of the selectivity of the feed-forward input are ρ_*E*_ = ρ_*I*_ = 0.06. The values of the background currents are *I*_*back, E*_ = 0.12 nA, *I*_*back, I*_ = 0.12 nA. The default parameters of the spike-timing dependent plasticity process are given by: *a*_+_ = 0.0128, *a*_−_ = −0.0045, τ_+_ = 30 ms, τ_−_ = 40 ms (Bi and Poo, 1998).

### The reduced model

The reduced model is composed by two populations of spiking neurons evenly spaced in the state space (0, 1]. The variable θ in this space denotes the preferred orientation of the external input divided by π. There are *N* neurons, half of them are excitatory and half of them inhibitory (Rosenbaum and Doiron, 2014). The location of the *k*th neuron is θ = *k*∕*N*. The input current to the *k*th excitatory (*A* = *E*) or inhibitory (*A* = *I*) neuron is given by:

(13) $$\begin{array}{l} I_{Ak}\left(t\right)=\overset{N}{\underset{j=1}{\sum }}g_{Ak,Ej}s_{E,j}\left(t\right)-g_{Ak,Ij}s_{I,j}\left(t\right)+i_{ext,Ak}\left(\theta \right), \end{array}$$

where *g*_*Ak, Bj*_ is the connection strength between neuron *j* in population *B* and neuron *k* in population *A* (*A, B* = *E, I*). $s_{A,j}\left(t\right)=\underset{l}{\sum }\delta \left(t-t_{A,j}^{l}\right)$ is the spike train of neuron *j* in population *A*. The *l*^*th*^ spike of neuron (*j, A*) occurs at time $t_{A,j}^{l}$. External input is provided by *i*_*ext, Ak*_(θ). The synaptic weights *g*_*Ak, Bj*_ are equal to the constant *g*_*AB*_ with probability *KC*_*AB*_(θ − ψ) or 0 else, where θ and ψ are the locations of neurons *k* and *j*, respectively. We take $C_{AB}\left(\theta -\psi \right)=\overset{\infty }{\underset{n=-\infty }{\sum }}c_{AB}\left(\theta -\psi -n\right)$ to insure periodic boundary conditions and we fix it in such a way that the average number of connections is always *K*, with 1 ≪ *K* ≪ *N*. Let us remark that this is the probability of having a connection in the state space, not in the physical space as in the previous model. A system with salt-and-pepper organization is represented by functions *C*_*AB*_(θ − ψ) that are totally flat. In contrast, a system with an orientation map has to be modeled with a modulated probability *C*_*AB*_(θ − ψ).

The synaptic weights are scaled according to:

(14) $$\begin{array}{l} g_{AB}=\frac{G_{AB}}{\sqrt{K}}, \end{array}$$

and the external inputs according to:

(15) $$\begin{array}{l} i_{ext,Ak}\left(\theta \right)=I_{ext,Ak}\left(\theta \right)\sqrt{K}. \end{array}$$

The mean firing rate of neuron *k* in the network is denoted by ν_*A*_(*x*) = 〈*s*_*A, k*_(*t*)〉, where <. > denotes average over time. Under these conditions, neuron *k* in population *A* will receive a total input given by:

(16) $$\begin{array}{l} \mu_{Ak}=\sqrt{K}\left[\underset{j}{\sum }\left(J_{Ak,Ej}\nu_{Ej}-J_{Ak,Ij}\nu_{Ij}\right)+I_{ext,Ak}\left(\theta \right)\right] \end{array}$$

where *J*_*Ak, Bj*_ = *G*_*AB*_*N*_*Ak, Bj*_ and *N*_*Ak, Bj*_ = 0, 1 is the number of connections between neuron *j* in population *B* and neuron *k* in population *A*. In the continuum limit, i.e., in the limit when the number of neurons *N* becomes very large, the interaction term can be written as *J*_*AB*_(θ, ψ) = *G*_*AB*_(*C*_*AB*_(θ − ψ) + ξ_*AB*_(θ, ψ)), where ξ_*AB*_(θ, ψ) are Gaussian random variables with 0 mean and correlation $<\xi_{AB}\left(\theta ,\psi \right)\xi_{A^{\prime }B^{\prime }}\left(\theta^{\prime },\psi^{\prime }\right)>=\delta_{AA^{\prime }}\delta_{BB^{\prime }}\delta \left(\theta -\theta^{\prime }\right)\delta \left(\psi -\psi^{\prime }\right)C_{AB}\left(\theta -\psi \right)∕K$. In the limit of large *K*, the interaction term becomes translational invariant (*J*_*AB*_(θ, ψ) = *J*_*AB*_(θ − ψ)) and the input can be written as:

(17) $$\begin{array}{l} \mu_{A}\left(\theta \right)=\sqrt{K}\left[J_{AE}*\nu_{E}\left(\theta \right)-J_{AI}*\nu_{I}\left(\theta \right)+I_{ext,A}\left(\theta \right)\right], \end{array}$$

where * stands for the circular convolution.

In order to have a finite result in the limit of large *K* the following equation has to be satisfied:

(18) $$\begin{array}{l} J_{AE}*\nu_{E}\left(\theta \right)-J_{AI}*\nu_{I}\left(\theta \right)+I_{ext,A}\left(\theta \right)=O\left(1∕\sqrt{K}\right) \end{array}$$

for *A* = *E, I*.

Taking the Fourier transform these integral equations become:

(19) $$\begin{array}{l} {\tilde{J}}_{AE}\left(n\right){\tilde{\nu }}_{E}\left(n\right)-{\tilde{J}}_{AI}\left(n\right){\tilde{\nu }}_{I}\left(n\right)+{Ĩ}_{ext,A}\left(n\right)=O\left(1∕\sqrt{K}\right), \end{array}$$

where $\tilde{f}(n)=\int_{0}^{1}f(\theta )\exp(-2i\pi n\theta )d\theta$. The solution of this linear system at the leading order is given by:

(20) $$\begin{array}{rcl} {\tilde{\nu }}_{E}\left(n\right) & = & \frac{{\tilde{J}}_{II}\left(n\right){Ĩ}_{ext,E}\left(n\right)-{\tilde{J}}_{EI}\left(n\right){Ĩ}_{ext,I}\left(n\right)}{{\tilde{J}}_{IE}\left(n\right){\tilde{J}}_{EI}\left(n\right)-{\tilde{J}}_{EE}\left(n\right){\tilde{J}}_{II}\left(n\right)} \end{array}$$

(21) $$\begin{array}{rcl} {\tilde{\nu }}_{I}\left(n\right) & = & \frac{{\tilde{J}}_{IE}\left(n\right){Ĩ}_{ext,E}\left(n\right)-{\tilde{J}}_{EE}\left(n\right){Ĩ}_{ext,I}\left(n\right)}{{\tilde{J}}_{IE}\left(n\right){\tilde{J}}_{EI}\left(n\right)-{\tilde{J}}_{EE}\left(n\right){\tilde{J}}_{II}\left(n\right)}. \end{array}$$

#### Stability of the balanced state

The balanced state solution from Equations (20) and (21) is stable only if the eigenvalues of the matrix:

(22) $$A(n)=\left|\begin{array}{cc} {\tilde{J}}_{EE}(n) & -{\tilde{J}}_{EI}(n)\\ {\tilde{J}}_{IE}(n) & -{\tilde{J}}_{II}(n) \end{array}\right|$$

have negative real part (Rosenbaum and Doiron, 2014). This condition, that is valid in the mean-field approximation to firing rate dynamics, implies that the following equations must be satisfied for each Fourier mode *n*:

(23) $$\begin{array}{rcl} {\tilde{J}}_{EE}\left(n\right)-{\tilde{J}}_{II}\left(n\right) & < & 0 \end{array}$$

(24) $$\begin{array}{rcl} {\tilde{J}}_{IE}\left(n\right){\tilde{J}}_{EI}\left(n\right)-{\tilde{J}}_{EE}\left(n\right){\tilde{J}}_{II}\left(n\right) & > & 0. \end{array}$$

## Results

### Orientation selectivity in the reduced model

We first analyze the selectivity properties of a one-dimensional network of spiking neurons in the balanced state. We assume that we are in the condition for which there is a stable solution with non-zero average firing rate. From Equations (20) and (21), this requires that (Rosenbaum and Doiron, 2014):

(25) $$\begin{array}{l} \frac{{Ĩ}_{ext,E}\left(0\right)}{{Ĩ}_{ext,I}\left(0\right)}>\frac{{\tilde{J}}_{EI}\left(0\right)}{{\tilde{J}}_{II}\left(0\right)}>\frac{{\tilde{J}}_{EE}\left(0\right)}{{\tilde{J}}_{IE}\left(0\right)}. \end{array}$$

Moreover, we will take the simple case where the spatial structure of the connectivity probability depends only on the presynaptic population, (${\tilde{J}}_{AB}\left(n\right)=G_{AB}{\tilde{J}}_{B}\left(n\right)$) and the external currents have the same spatial profile in both populations (*Ĩ*_*ext, A*_(*n*) = *Ĩ*_*ext*_(*n*)*I*_*ext, A*_). In that situation, Equations (20) and (21) become:

(26) $$\begin{array}{rcl} {\tilde{\nu }}_{E}\left(n\right) & = & \frac{\left(G_{II}I_{ext,E}-G_{EI}I_{ext,I}\right){Ĩ}_{ext}\left(n\right)}{\left(G_{IE}G_{EI}-G_{EE}G_{II}\right){\tilde{J}}_{E}\left(n\right)} \end{array}$$

(27) $$\begin{array}{rcl} {\tilde{\nu }}_{I}\left(n\right) & = & \frac{\left(G_{IE}I_{ext,E}-G_{EE}I_{ext,I}\right){Ĩ}_{ext}\left(n\right)}{\left(G_{IE}G_{EI}-G_{EE}G_{II}\right){\tilde{J}}_{I}\left(n\right)}. \end{array}$$

The feed-forward input current on the neuron located at coordinate θ when the stimulus orientation is θ_0_ will be taken as:

(28) $$\begin{array}{l} I_{ext,A}\left(\theta ,\theta_{0}\right)=\left(1+2\rho \cos\left(2\pi \left(\theta -\theta_{0}\right)\right)\right)I_{ext,A}. \end{array}$$

Let us remark that the state space does not necessarily correspond with the physical space. For a salt-and-pepper organization they are, in fact, independent: The physical location of a neuron is unrelated to its preferred orientation. This means that the connection probabilities must have a flat spatial structure.

According to Equation (28) and using the definition $\tilde{f}(n)=\int_{0}^{1}f(\theta )\exp(-2i\pi n\theta )d\theta$, the following result can be obtained: *Ĩ*_*ext*_(0) = *I*_*ext, A*_, *Ĩ*_*ext*_(1) = ρ*I*_*ext, A*_ and *Ĩ*_*ext*_(*n*) = 0 for all *n* > 1. Therefore, ρ is simply the orientation selectivity index of the external current (see Equation 7). According to Equations (26) and (27), ${\tilde{\nu }}_{E}\left(n\right)={\tilde{\nu }}_{I}\left(n\right)=0$ for all *n* > 1 while:

(29) $$\begin{array}{rcl} \frac{{\tilde{\nu }}_{E}\left(1\right)}{{\tilde{\nu }}_{E}\left(0\right)} & = & \rho \frac{{\tilde{J}}_{E}\left(0\right)}{{\tilde{J}}_{E}\left(1\right)} \end{array}$$

(30) $$\begin{array}{rcl} \frac{{\tilde{\nu }}_{I}\left(1\right)}{{\tilde{\nu }}_{I}\left(0\right)} & = & \rho \frac{{\tilde{J}}_{I}\left(0\right)}{{\tilde{J}}_{I}\left(1\right)}. \end{array}$$

This result implies that the selectivity of the cortical activity ($\frac{{\tilde{\nu }}_{A}\left(1\right)}{{\tilde{\nu }}_{A}\left(0\right)}$) is directly proportional to the selectivity of the feed-forward input (ρ) but inversely proportional to the spatial modulation of the intracortical connections (${\tilde{J}}_{E}\left(1\right)$). This somehow surprising behavior was already discussed in van Vreeswijk and Sompolinsky (2005). The cortical network receives an excitatory input of order $\sqrt{K}$ that has to be canceled by the intracortical activity giving rise to a net input of order 1. The intracortical input to a given neuron is a convolution of the cortical activity and the connectivity matrices. Then, if the connectivity matrices have a wide spatial profile, the cortical activity must be narrower to keep a fixed spatial profile of the intracortical input. This leads to strong cortical selectivity since it contributes to the term $\tilde{\nu }\left(1\right)$ of Equations (29) and (30). This result is in contrast with the one found in Ben-Yishai et al. (1995), where the modulation of the excitatory connections leads to increasing selectivity. The difference is due to the fact that in this last case the network is not in a balanced state. Thus, the recurrent connections do not lead to the cancelation of the leading term of the input but, instead, to its amplification.

#### Comparison of orientation selectivity in salt-and-pepper and orientation map

For a given feed-forward input, the selectivity of the cortical activity is determined by the ratio $\frac{{\tilde{J}}_{A}\left(0\right)}{{\tilde{J}}_{A}\left(1\right)}$. For both structures (salt-and-pepper and orientation map) the numerator is equal to the average number of intracortical connections *K*. In contrast, the behavior of the denominator is completely different.

For the salt-and-pepper structure the connection probability is, on average, independent of the distance in the state space. According to this, selectivity should be infinite in the limit of large *K* (in that limit ${\tilde{J}}_{A}\left(1\right)=0$), but this is impossible because selectivity cannot be larger than 1. However, as fluctuations of the connectivity patterns generate contributions to the selectivity that are proportional to $1∕\sqrt{K}$ (see Supplementary Material), and these contributions become dominant for very weakly modulated interactions, the selectivity remains bounded between 0 and 1. In practice, numerical simulations must be performed in order to test whether a reasonable value of connectivity is compatible with the balanced state. In this scenario, the balanced state can be preserved in the limit of large *K* if the modulation of the input, ρ, also scales as $1∕\sqrt{K}$. This is studied in Hansel and van Vreeswijk (2012).

In contrast, in systems with an orientation map the numerator and the denominator are both proportional to *K*. As a consequence, for large connectivity, and keeping the rest of the parameters the same, one should expect salt-and-pepper structures to be more selective than systems with an orientation map, as the denominator of orientation map (~*K*) is larger than the denominator of salt-and-pepper (($\sim 1∕\sqrt{K}$)). Moreover, for systems with an orientation map, selectivity could increase by taking a broader connection probability profile.

#### Effect of plasticity on orientation selectivity in the reduced model

The plasticity rules of Equations (10) and (11) depend on the precise timing of the pre- and post-synaptic spikes. However, in the balanced state, significant cross-correlations between different spike trains are not expected (van Vreeswijk and Sompolinsky, 1998; Renart et al., 2010). In this situation, the net change in the synaptic strength will be controlled by the total number of spikes, by the integral of the STDP window and by the synaptic strength itself (through the terms *w*_*iA, jE*_ and 2 − *w*_*iA, jE*_ in Equations 10, 11). As the normalized value of the synapses is initially *w*_*iA, jE*_ = 1, the integral is equal to α = *a*_+_ τ _+_ + *a*_−_ τ _−_. In order to estimate the effect of plasticity on selectivity we neglect the effect of the non-linear terms in the plasticity rule. Thus, the change in the synaptic strength per unit time between neurons (*j, E*) and (*k, A*), *A* = *E, I*, is given by:

(31) $$\begin{array}{l} \Delta w_{Ak,Ej}=\alpha \nu_{Ak}\nu_{Ej}. \end{array}$$

If the feed-forward input is described by Equation (28) and we neglect the changes of the firing rates generated by the changes in the synaptic weights, the firing rates can be approximated as a function of the stimulus orientation θ:

(32) $$\nu_{Ak}(\theta )=\nu_{Ak}^{{}^{\circ}}(1+2\xi_{Ak}\cos(2\pi (\theta -\theta_{Ak}))$$

(33) $$\nu_{Ej}(\theta )=\nu_{Ej}^{{}^{\circ}}(1+2\xi_{Ej}\cos(2\pi (\theta -\theta_{Ej}))$$

where θ_*Ak*_, θ_*Ej*_ are the preferred orientations, $\nu_{Ak}^{{}^{\circ}}$, $\nu_{Ej}^{{}^{\circ}}$ are the firing rates averaged over the stimulus orientations and ξ_*Ak*_, ξ_*Ej*_ are the selectivities of neurons (*k, A*) and (*j, E*), respectively. Notice that the approximation will be valid only when the changes of the synaptic weights are small. Replacing Equations (32), (33) in Equation (31) and averaging over the stimulus orientation we obtain:

(34) $$\bar{\Delta w_{Ak,Ej}}=\alpha \nu_{Ak}^{{}^{\circ}}\nu_{Ej}^{{}^{\circ}}(1+2\xi_{Ak}\xi_{Ej}\cos(2\pi (\theta_{Ak}-\theta_{Ej})).$$

A similar result is obtained for a fixed stimulus orientation after averaging over θ_*Ak*_ and θ_*EJ*_, keeping θ_*Ak*_ − θ_*EJ*_ constant, and assuming that the mean rates and the selectivities of the neurons are independent from the position on the network. This means that the specific protocol for the stimulus presentation does not affect the synaptic weights.

For the salt-and-pepper architecture this result implies the generation of functional connectivity, i.e., a connectivity pattern that is linked to the relative preferred orientation of the neurons. For positive values of α, neurons with similar preferred orientations will have stronger synapses than neurons with orthogonal preferred orientations. In terms of the Fourier components of the connectivity matrices, the first Fourier component will be increased by a term that is proportional to the square of the selectivity of the cortical activity. But, at the same time, the zeroth Fourier component will be reinforced by a term that is order 1 in the selectivity. Thus, for initial small values of cortical selectivity, plasticity will improve it. It is interesting to note that this improvement does not emerge because of the generation of functional connectivity, but in spite of it.

For systems with cortical maps the situation is similar: the modulated component of the connectivity matrices will be reinforced, but typically less than the non-modulated part. This mechanism leads to an increment of the selectivity.

Let us remark that these conclusions are valid only at the initial stages of learning, where the non-linear terms can be neglected. The long term behavior must be analyzed on the network model with numerical simulations.

### Effect of reconnection probability on orientation selectivity in the reduced model

For the salt-and-pepper structure with a connection probability that depends only on the physical distance between the neurons, the connection probability in the space of preferred orientations is totally flat, i.e.,

(35) $$\begin{array}{l} p_{iA,jB}=\frac{K}{N∕2}, \end{array}$$

so that we always have in average *K* connections per neuron for each one of the *N*∕2 neurons in population *A* incoming from population *B* (*A, B* = *E, I*). The reconnection probability rule of Equation (12) gives rise to modulation of the probability in this state space. If the preferred orientations of the neurons are perfectly correlated with the orientations that maximize the feed-forward input the probability of connection between neuron (*i, A*) and (*j, B*) will be:

(36) $${\tilde{p}}_{iA,jB}=\frac{K}{N/2}(1+\epsilon_{cB}\cos(2\pi (\theta_{jB}-\theta_{iA})).$$

This implies that *C*_*AB*_(θ − ψ) = 1 + ϵ_*cB*_cos(2π(θ − ψ)) and (in the limit of large *K*) that *J*_*AB*_(θ − ψ) = *G*_*AB*_(1 + ϵ_*cB*_cos(2π(θ − ψ))). The first Fourier components are:

(37) $$\begin{array}{rcl} {\tilde{J}}_{AE}\left(1\right) & = & G_{AE}\epsilon_{cE}∕2 \end{array}$$

(38) $$\begin{array}{rcl} {\tilde{J}}_{AI}\left(1\right) & = & G_{AI}\epsilon_{cI}∕2, \end{array}$$

while the other Fourier components are not affected by the reconnection. By replacing Equations (37) and (38) into Equations (29) and (30) we can see that more functional modulation implies a reduction of the orientation selectivity:

(39) $$\begin{array}{rcl} \frac{{\tilde{\nu }}_{E}\left(1\right)}{{\tilde{\nu }}_{E}\left(0\right)} & \propto & \frac{1}{\epsilon_{cE}} \end{array}$$

(40) $$\begin{array}{rcl} \frac{{\tilde{\nu }}_{I}\left(1\right)}{{\tilde{\nu }}_{I}\left(0\right)} & \propto & \frac{1}{\epsilon_{cI}}. \end{array}$$

We can also observe by replacing Equations (37) and (38) into Equation (23) that changes in functional connectivity may lead to the loss of stability of the balanced state. This will happen when:

(41) $$\begin{array}{l} \frac{\epsilon_{cE}}{\epsilon_{cI}}>\frac{G_{II}}{G_{EE}}, \end{array}$$

since Equation (23) will not be satisfied for *n* = 1. This means that the excitatory modulated recurrent input is too strong to be compensated by the inhibitory–inhibitory interactions. Under these conditions, the predictions of Equations (29) and (30) are not valid anymore and numerical simulations are required to assess selectivity.

### Orientation selectivity in the network model

**Figure 3:**
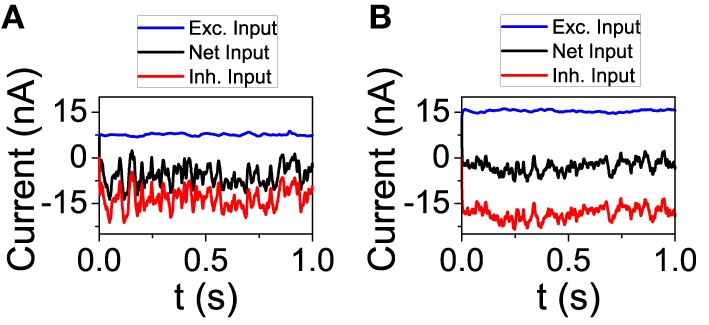
**The excitatory and inhibitory inputs to one neuron tend to cancel even as they grow stronger with larger connectivity**. **(A)** *K* = 250, **(B)** *K* = 1000. *N*_*E*_ = 16129, *N*_*I*_ = 4096. The rest of the parameters are set to default.

We now analyze the behavior of the network model in order to check some of the predictions done by the reduced model. First, we studied the dynamical state from the point of view of the total input on an individual neuron. We performed simulations with connectivity *K* = 250 and 1000. For each value of *K*, we chose a neuron and depicted its excitatory and inhibitory input (see Figure 3). On one hand, as the theory of balanced state predicts, as connectivity grows both contributions to the total input increase proportionally to $\sqrt{K}$. On the other hand, the net result is almost the same, showing the cancelation between the two components. The average firing rate of the excitatory population has changed from *f*_*E*_ = 3.02 Hz for *K* = 250 to *f*_*E*_ = 2.61 Hz for *K* = 1000. For the inhibitory population, the firing rate has gone from *f*_*I*_ = 7.81 Hz to *f*_*I*_ = 5.71 Hz.

**Figure 4:**
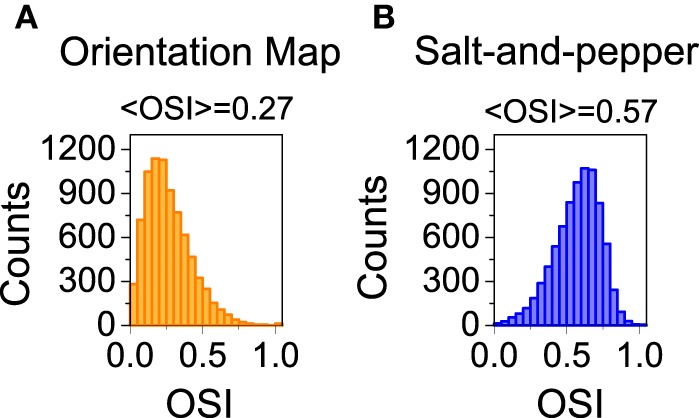
**Distribution of orientation selectivity index for the neurons of the excitatory population**. Systems with orientation maps **(A)** are less selective than networks with a salt-and-pepper organization **(B)**. Parameters set to default values.

We also studied the selectivity properties of the two cortical architectures mentioned above: orientation map and salt-and-pepper. For the system with an orientation map, we found that the mean orientation selectivity index of the excitatory population is < *OSI*_*E*_ > = 0.27 (see Figure 4A). If exactly the same network is considered, but now it has a salt-and -pepper structure instead of an orientation map, < *OSI*_*E*_ > = 0.57 (see Figure 4B). Note that the change in selectivity appears without a strong change in the average firing rate, that has gone from *f*_*E*_ = 2.51 Hz (orientation map) to *f*_*E*_ = 2.62 (salt-and-pepper). This is a somehow counterintuitive result, but it agrees with the predictions of the reduced model. In systems with an orientation map, the correlation between the position of the neurons in the network and their preferred orientations gives rise to functional connectivity. Therefore, there will be a significant modulation of the connectivity matrix in the functional space, that will reduce selectivity according to Equations (29) or (30). This does not necessarily imply that systems with salt-and-pepper organization should always have more selectivity than systems with orientation map. This could only be the case if all the other parameters are kept constant. For instance, the modulation of the external input ρ. A given value of orientation selectivity in layer 2/3 will require a more modulated input in a system with an orientation map than in a network with a salt-and-pepper structure.

#### Effect of stdp on orientation selectivity in the network model

**Figure 5:**
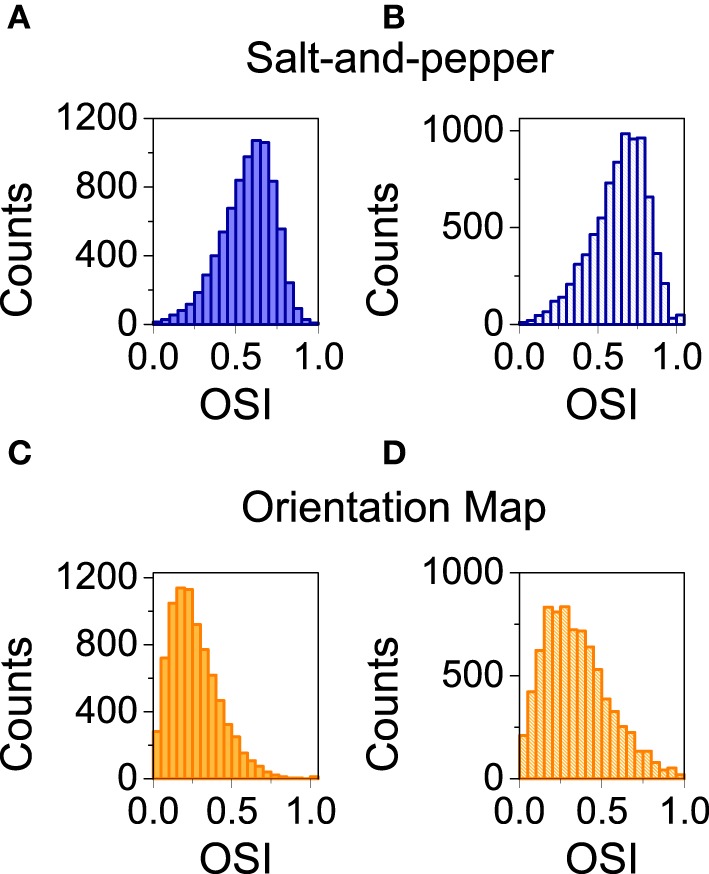
**Spike-timing dependent plasticity increases selectivity**. **(A)** Salt-and-pepper before synaptic modifications < *OSI* > = 0.57, **(B)** salt-and-pepper after synaptic modifications < *OSI* > = 0.63, **(C)** orientation map before synaptic modifications < *OSI* > = 0.27, **(D)** orientation map after synaptic modifications < *OSI* > = 0.36. In all the cases we show the distribution of orientation selectivity index for the neurons of the excitatory population. Parameters as in the previous figure.

Here, we analyze the influence of STDP on orientation selectivity for both architectures (salt-and-pepper and orientation map). In Figure 5 we show the distribution of OSI of the neurons in the excitatory population. In both cases, the average selectivity increases once plasticity is applied, although in a much smaller degree in salt-and-pepper (< *OSI* > from 0.57 to 0.63) than in orientation map (< *OSI* > from 0.27 to 0.36). We checked that these values are stable by comparing simulations of 20 s and 40 s long.

**Figure 6:**
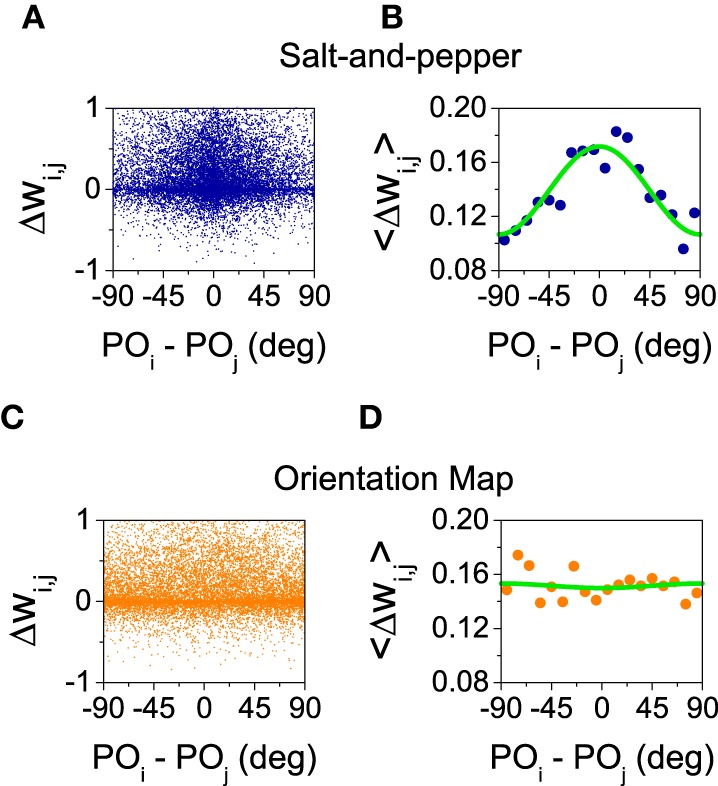
**Only for the system with salt-and-pepper organization STDP gives rise to a significant degree of functional connectivity**. **(A)** Final value of the normalized synaptic efficacy *w*_*i, j*_ (see Equations 10 and 11) between neurons (*i, E*) and (*j, E*) as a function of the difference in the preferred orientations of the same neurons. **(B)** Average of the previous points in intervals of 10°. Green line: fit with the function $F\left(x\right)=a_{0}+2a_{1}\cos\left(\pi x∕90^{{}^{\circ}}\right)$. The values of the fitted parameters are *a*_0_ = 0.14, *a*_1_ = 0.016. **(C,D)** As in **(A,B)** but for the orientation map. The values of the fitted parameters are now *a*_0_ = 0.15, *a*_1_ = −0.00084. All the rest of parameters as in the previous figure.

What is the reason for the different behaviors? In the balanced state, the change in selectivity is controlled by the ratio between the mean value (${\tilde{J}}_{A}\left(0\right)$) and the modulation $\left({\tilde{J}}_{A}(1)\right)$) of the connectivity matrices in the orientation space (see Equations 29 and 30). These two quantities can be estimated from the simulations by evaluating the change in the synaptic strength as a function of the difference of the preferred orientations between the pre- and post-synaptic neurons. Those changes are shown in Figure 6 for salt-and-pepper (Figures 6A,B) and orientation map (Figures 6C,D).

Figures 6A,C show how the strength of the connections changes as a function of the difference of the preferred orientations. As those changes display a great amount of variability, in Figures 6B,D we show the average for all the pairs of neurons whose differences in preferred orientations fall within an interval of 10 degrees (i.e., [0°, 10°), [10°, 20°), …). The resulting averages were fitted with the function $a_{0}+2a_{1}\cos\left(\pi x∕90^{{}^{\circ}}\right)$. The value of *a*_0_ gives an estimation of the change in the mean synaptic strength $\Delta {\tilde{J}}_{A}\left(0\right)$, while *a*_1_ approximates the change in the modulation $\Delta {\tilde{J}}_{A}\left(1\right)$. In both cases *a*_0_ is positive (see Figure 6), this means the connections are strengthened in presence of STDP. For the orientation map the change in the modulation is very small, what leads to a significant improvement of selectivity as the numerator of Equation (29) grows much more as compared with the denominator. For the salt-and-pepper structure both the mean value and the modulation of the synaptic strength grow significantly. This modification gives rise to opposite contributions to the selectivity: While the increase of the mean value tends to improve selectivity, the strong modulation reduces it. For this particular case, the net effect is small but positive, although we cannot rule out a situation where the net result is negative.

#### Effect of reconnection probability on orientation selectivity in the salt-and-pepper structure

As presented here, the spike-timing dependent plasticity rule can either strengthen or weaken existing connections but cannot create or destroy existing ones (although destruction could be implemented by including an absorbing barrier for a zero value of the synapse). Experimental results (Ko et al., 2013) indicate that a substantial reconnection process takes place in the initial stage of development in rodents. After eye opening, local connectivity reorganizes extensively in such a way that new connections between neurons with similar visual responses arise. However, connectivity rate does not significantly change during this process and only a modest increment in orientation selectivity takes place, taking average OSI from 0.62 to 0.68 (Ko et al., 2013). Another motivation for introducing a reconnection mechanism as implemented in Equation (12), is that the average synaptic efficacy is automatically kept constant. In the balanced state the mean firing rates are determined by the mean connectivity (see Equations 26, 27 for *n* = 0). As the reconnection process does not affect the total number of connections, there should be no change in the average firing rates. Thus, any change in the selectivity properties will be given by a modification of the modulated part of the tuning curves.

**Figure 7:**
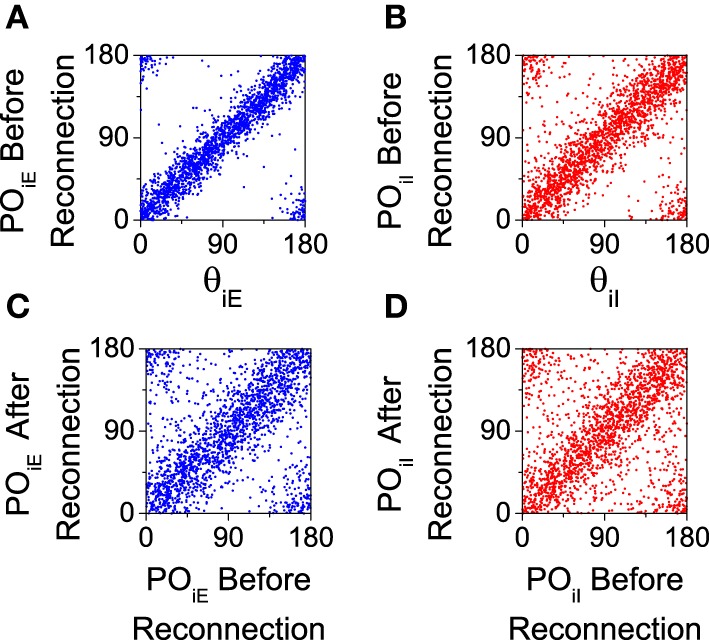
**Preferred orientations of the neurons (*PO*_*iA*_) are strongly correlated to preferred orientations of the feed-forward inputs (θ_*iA*_) and they are conserved after reconnection**. All the graphs correspond to the salt-and-pepper organization with default parameters. **(A,C)** Excitatory neurons. **(B,D)** Inhibitory neurons. In all the panels we show 2025 neurons. Parameters set as default.

We analyze the effect of this reconnection process in our computational model. We first calculate the preferred orientation of each neuron using the population vector (Equation 8) with a simulation of 20 s. Then, according to that preferred orientation, the connection probability is recalculated (Equation 12) and a new connectivity matrix is generated. Note that even for the salt-and-pepper organization this process will generate a new network with a significant degree of functional connectivity. This procedure will work only if there is a significant degree of correlation between the preferred orientations both before and after the reconnection. This is verified for both populations in Figure 7, where we show that the preferred orientations are strongly correlated with the preferred orientation of the feed-forward input (see Figures 7A,B), and that the are mostly preserved by the reconnection process (see Figures 7C,D).

**Figure 8:**
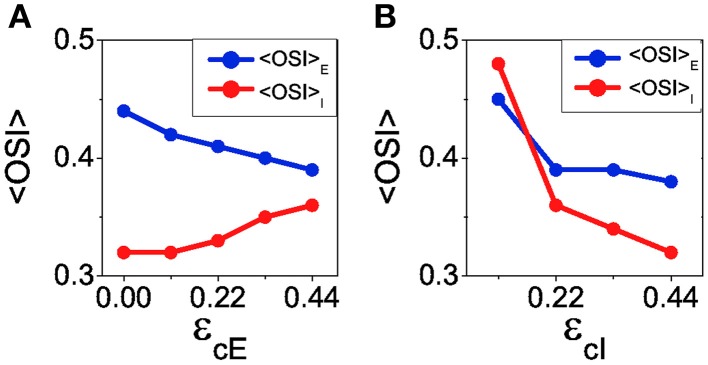
**When the balanced state is stable increasing functional connectivity in the excitatory interactions leads to a loss of selectivity in the excitatory population and an increase of selectivity in the inhibitory population**. In contrast, increasing functional connectivity in the inhibitory interactions always leads to reduction of selectivity. **(A)** Average orientation selectivity index for the excitatory population (blue) and for the inhibitory population (red) as a function of the reconnection parameter ϵ_*cE*_ keeping ϵ_*cI*_ = 0.22. **(B)** The same averages as a function of ϵ_*cI*_ with ϵ_*cE*_ = 0.44.

The reconnection parameters (ϵ_*cE*_, ϵ_*cI*_) determine how much modulated the corresponding connectivity matrix is. It is important to remark that if the degree of modulation in the excitatory interactions is too large as compared to the modulation in the inhibitory interactions, the balanced state might become unstable (see Equation 41). The results of Ko et al. (2013) indicate a degree of functional connectivity between excitatory neurons compatible with a value of ϵ_*cE*_ ≈ 0.44 (see Figure 2i in Ko et al., 2013). According to the coupling values we are using, and asking for the network to remain in the stable balanced state, ϵ_*cI*_ is required to be at least equal to 0.11 (because |*G*_*EE*_ ∕*G*_*II*_| = 1∕4, see Equation 41). In Figure 8 we show the mean values of selectivity of both populations for combinations of ϵ_*cE*_, ϵ_*cI*_ that preserve the stability of the balanced state. We observe that increasing the functional connectivity in the excitatory population leads to a decrease in the excitatory selectivity (Figure 8A), while increasing the functional connectivity in the inhibitory population conveys a reduction in the inhibitory selectivity (Figure 8B); Both effects were predicted by the reduced model. Note, however, that in both cases there is a change of selectivity for the population whose interactions have not been affected by the reconnection process (population *I* in Figure 8A and population *E* in Figure 8B). This behavior is not predicted by the reduced model and deserves further investigation to fully understand it. It is probably due to the different topology between the two models. Let us note that in all the cases, the reconnection rule generates a change in the mean firing rates always smaller than 5%.

**Figure 9:**
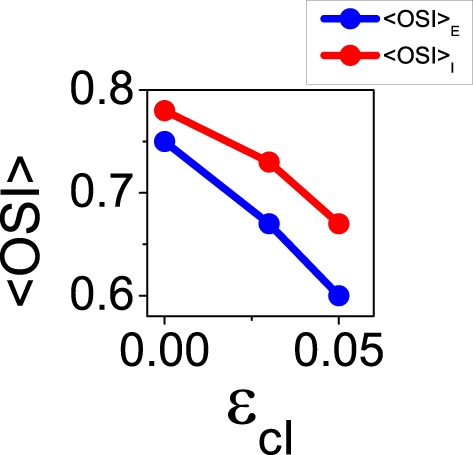
**When the balanced state is unstable for the first Fourier mode, increasing functional connectivity in the inhibitory interactions leads to reduction of selectivity**. Average orientation selectivity index for the excitatory population (blue) and for the inhibitory population (red) as a function of ϵ_*cI*_ with ϵ_*cE*_ = 0.44.

It is also possible to choose a combination of the reconnection parameters so that the balanced state becomes unstable, for instance ϵ_*cE*_ = 0.44 and ϵ_*cI*_ = 0.03. Using those parameters, the mean value of orientation selectivity is < *OSI*_*E*_ > = 0.67 (see Figure 9). This result represents an increment of selectivity from the control situation, at which < *OSI*_*E*_ > = 0.57. This increase cannot be predicted by the theory of the reduced model in the balanced state, since the network is not balanced anymore. Note that the loss of stability for the first Fourier mode does not lead to a significant change of the mean firing rates, that are given by *f*_*E*_ = 2.38 Hz and *f*_*I*_ = 6.19 Hz for ϵ_*cE*_ = 0.44 and ϵ_*cI*_ = 0.03 (compared to *f*_*E*_ = 2.61 Hz and *f*_*I*_ = 6.05 Hz in the control case). As the balanced state is not longer stable, the results we show here may depend on the details of the network dynamics since the non-linearities of the neuron transfer curve are not washed out by the balanced equations anymore.

## Discussion

We studied the selectivity properties of systems with orientation map or salt-and-pepper structure. We found that, keeping all the rest of the parameters the same, systems with an orientation map were less selective for orientation than systems with a salt-and-pepper organization. If we define functional connectivity as the correlation between the connectivity structure and the responses of the neurons, the result we found is due to the fact that the more functional connectivity the system has, the more modulated its connectivity profile is. Therefore, according to Equations (29) and (30), the system becomes less selective to orientation. This does not mean that systems with salt-and-pepper architecture should always be more selective than systems with an orientation map. For instance, high selectivity in a system with an orientation map can be achieved by increasing the selectivity of the feed-forward input.

We worked in the framework of balanced networks. Several experimental works support the idea that cortical systems operate in a balanced regime, i.e., excitation is compensated by inhibition (Destexhe et al., 2003; Shu et al., 2003; Haider et al., 2006). This idea was proposed to explain why neurons *in vivo* fire so irregularly (Holt et al., 1996) in spontaneous as well as in sensory-evoked activity (van Vreeswijk and Sompolinsky, 1996, 1998). It also intends to explain the highly irregular neuronal firing pattern in persistent activity by introducing synaptic non-linearities (Hansel and Mato, 2013).

For a network to operate in the balanced regime, the recurrent and the feed-forward inputs have to be large as compared to the threshold of the neurons (van Vreeswijk and Sompolinsky, 1996, 1998; Hansel and van Vreeswijk, 2012). Under very general conditions, the balance emerges from the recurrent dynamics of the network so that the net input is much smaller than its excitatory and inhibitory components. Thus, as the mean value of the net input is below the threshold neurons fire because of the fluctuations of the input. Therefore, the firing properties are essentially determined by the statistical properties of the input and the intrinsic dynamic of the neuron is not relevant (Hansel and van Vreeswijk, 2002). Moreover, in Rauch et al. (2003) it was found that the firing properties of cortical neurons *in vivo* can be well approximated by an integrate-and-fire model. These results imply that the findings we here present do not depend on the integrate-and-fire model, but they apply to other neuron models too. However, at high firing rates this conclusion does not longer hold because of the refractory period of the neurons, but these effects are irrelevant for systems that are as far from the saturation of the firing rate as our results are.

In the balanced state the main role of the recurrent connections is to cancel the leading order of the feed-forward input. One of the consequences of this behavior is that increasing all the coupling constants by a given factor leads to a decrease in the firing rates by the same factor if the external inputs are kept constant (van Vreeswijk and Sompolinsky, 1998). As at the population level the system is linear, the same behavior is found for each one of the Fourier components of the activity (see Equations 26 and 27). In other words, there is an inverse relation between the modulation of the activity profile and the modulation of the connectivity profile. This fact was already observed in van Vreeswijk and Sompolinsky (2005), where it was analyzed in terms of the cancelation of the leading terms of the external input. In the limit of large size the recurrent input is equal to the convolution between the network activity and the connectivity matrices. Therefore, in order to keep the recurrent input constant, wider connectivity profiles and narrower activities must be balanced out. This counterintuitive behavior is not always observed nor well understood. For instance, in Corey and Scholl (2012), it was stated that increasing the connection probability of nearby neurons with similar orientation preference might further enhance their selectivity. However, here we show that this is not necessarily true. In fact, for networks in the balanced state the opposite result is the one that should be expected.

In a recent work (Ko et al., 2013) it was shown that after eye opening, local connectivity reorganized extensively without changing the overall connectivity. This means that connections between neurons with similar visual response were created and strengthened, but that the mean value of connections remained the same. This reconnection process must coexist with plasticity mechanisms that affect the synaptic strength without changing the number of synapses. Such a plasticity mechanism could be STDP that, in fact, has also been found in cortical systems (Markram et al., 1997; Feldman, 2000).

We investigated both mechanisms separately: The STDP process and the reconnection rule. Regarding STDP, this rule leads to a significant increment in the orientation selectivity for systems with orientation map (~50%), while it results in a very small effect for salt-and-pepper (~10%). We also found that in the first case the plasticity rule did not generate significant functional connectivity, while in the second one, it did. The observed change in selectivity is consistent with the one found with the theoretical analysis of the reduced model. If there is a positive effect on the selectivity it is because the mean value of the connections increases more than its modulation. For instance, as for the salt-and-pepper structure STDP generates a strong modulation of the connectivity profile, the generation of functional connectivity results in a negative effect on selectivity. On the other hand, for systems with an orientation map the most significant effect of STDP is the increment of the average connectivity without giving rise to functional connectivity. This combination strongly increases selectivity. Note that despite the fact that STDP can generate functional connectivity, this feature does not contribute to an increment of selectivity. The rise in selectivity is dominated by the change of the mean value of the connectivity profile as compared to the modulation that it acquires after plasticity is applied. This non-trivial result is a consequence of the dynamics of the balanced regime.

It is important to remark that the difference in the growth of selectivity for both architectures that we found, is probably due to the small degree of selectivity that systems with an orientation map have in the first place in our simulations. For the STDP rule, the amount of functional connectivity generated is proportional to the square of the selectivity before learning (see Equation 34). As the initial value of selectivity in salt-and-pepper is more than twice as large as the original value of selectivity in orientation map, much more functional connectivity is generated in the first case. However, note that some kind of self-regulatory mechanism arises: Systems with strong selectivity develop more functional connectivity as STDP is applied, but the more functional connectivity is generated, the less the selectivity increases. In other words, for systems that are highly selective to orientation, STDP does not produce such an increment of selectivity as it does for systems that are initially less selective.

Though in some systems inhibitory connections exhibit STDP (Haas et al., 2006) too, for simplification we here assume that only excitatory synapses are plastic. However, the effect of plasticity on inhibitory connections can be easily analyzed with the same tools presented here. For instance, an increase of the modulated part of the inhibitory interactions should lead to some degree of reduction of the orientation selectivity in the inhibitory population if the non-modulated part is kept constant (see Equation 30). An increase of the non-modulated part, in contrast, should lead to more selectivity. These effects are the same as the ones found for the excitatory interactions. The difference between both of them is that an increment of the modulation of the inhibitory interactions cannot lead to the loss of the stability of the balanced state. If Equation (23) is initially satisfied, then an increase in the modulation would only make the second term of the left side more negative.

Similarly, the effect of homeostatic processes (Turrigiano and Nelson, 2000, 2004) that tend to keep constant the mean firing rate could be analyzed too. These mechanisms might affect selectivity even if the total amount of modulation of the tuning curves is kept constant. For instance, an additive homeostatic process would modify the zeroth Fourier component of the connectivity matrices but not the other ones. If this mechanism acts at the same time as STDP on the excitatory interactions, it will cancel the change of the mean value of the connections while keeping constant the rise of the modulated part. This will lead to the loss of orientation selectivity. In contrast, a global synaptic rescaling that equalizes the postsynaptic firing rates (Turrigiano, 1999; van Rossum et al., 2000) will affect by the same factor all the synapses independently of the position of the neurons in the state space. Therefore, the ratio between the zeroth Fourier component and the first one will not be affected and the change of selectivity will be the same as without homeostasis.

Finally, we want to remark that in order to preserve the stability of the balanced state, some constraints between the modulation of the excitatory interactions and the modulation of the inhibitory interactions must be satisfied (see Equation 56). If the modulation of the excitatory interactions is large enough, then the balanced state becomes unstable for the first Fourier mode. Remarkably, the loss of stability does not give rise to a significant change of the average firing rate, implying that the instability does not affect the zeroth Fourier mode. This comment is also applicable to the generation of functional connectivity using STDP only on the excitatory interactions: Increasing the modulation of the excitatory population gives rise to an increase in selectivity, but even a small increment of the modulation of the inhibitory interactions might cancel the effect. This could explain the results of Ko et al. (2013), where it was found that a significant increase in functional connectivity of the excitatory interactions generates a very small change of orientation selectivity. In any case, it is clear that more information on the functional connectivity of the inhibitory interactions is needed to understand completely the behavior of the system.

### Conflict of interest statement

The authors declare that the research was conducted in the absence of any commercial or financial relationships that could be construed as a potential conflict of interest.

## Supplementary material

Sample software codes implemented in C are available at: http://fisica.cab.cnea.gov.ar/estadistica/matog/v1-stdp/software.html
