# Supplementary material for: The effect of synaptic plasticity on orientation selectivity in a balanced model of primary visual cortex
Source: Front Neural Circuits. 2015 Aug 20;9:42. doi: 10.3389/fncir.2015.00042 (PMC4542321; doi:10.3389/fncir.2015.00042)
Supplement: Supplementary file 1 [file DataSheet1.PDF]

Supplementary Material

Keeping the terms coming from the fluctuations in the connectivity patterns Eq. 17 becomes

$$\begin{aligned}\mu_A(\theta) &= \sqrt{K}[J_{AE} * \nu_E(\theta) - J_{AI} * \nu_I(\theta) + I_{ext,A}(\theta) + \\ &\int_0^1 dw_{AE,\psi} G_{AE} \xi_{AE,\psi}(\theta) \nu_E(\psi) - dw_{AI,\psi} G_{AI} \xi_{AI,\psi}(\theta) \nu_I(\psi)].\end{aligned}$$

where $w_{AB,\psi}$ are Wiener processes and $\xi_{AB,\psi}(\theta)$ are Gaussian white noises with 0 mean. Their variances are given by $< \xi_{AB,\psi}(\theta)^2 >= C_{AB}(\psi - \theta)/K$. Using

$$\nu_A(\psi) = \sum_m \tilde{\nu}_A(m) \exp(2im\psi)$$

we obtain

$$\begin{aligned}\tilde{\mu}_A(n) &= \sqrt{K}[\tilde{J}_{AE}(n)\tilde{\nu}_E(n) - \tilde{J}_{AI}(n)\tilde{\nu}_I(n) + \tilde{I}_{ext,A}(n) + \\ &\sum_m R_{AE}(n,m)\tilde{\nu}_E(m) - R_{AI}(n,m)\tilde{\nu}_I(m)],\end{aligned}$$

where

$$R_{AB}(n,m) = G_{AB} \int_0^1 dw_{AB,\psi} dw_{AB,\theta} \exp(2i(m\psi - n\theta)) \sqrt{\frac{C_{AB}(\psi - \theta)}{K}}.$$

According to this, $R_{AB}(n,m)$ are random variables with 0 mean and correlation

$$< R_{AB}(n,m) R_{A'B'}(n',m') >= \delta_{AA'} \delta_{BB'} \delta_{m+m',n+n'} \frac{\tilde{C}_{AB}^{1/2}(n) \tilde{C}_{AB}^{1/2}(n')}{K}.$$

According to this we have

$$\tilde{\mu}_A(n) = \sqrt{K}[(\tilde{J}_{AE}(n) + R_{AE}(n,n))\tilde{\nu}_E(n) - (\tilde{J}_{AI}(n) + R_{AI}(n,n))\tilde{\nu}_I(n) + \tilde{I}_{ext,A}(n)]. \tag{47}$$

For the "salt-and-pepper" organization we have $\tilde{J}_{AB}(0) \neq 0$ and $\tilde{J}_{AB}(n) = 0$ for $n \neq 0$. Let us consider the case where $\tilde{I}_{ext,A}(n) = O(1)$ for all n. For $n = 0$ we recover the standard balance equations for which $\tilde{\nu}_A(0) = O(1)$.
